# Mitochondrial genome of *Tamarixia radiata* (Hymenoptera: Chalcidoidea: Eulophidae) and phylogenetic analysis

**DOI:** 10.1080/23802359.2019.1660259

**Published:** 2019-09-03

**Authors:** Yimin Du, Xiang Song, Xinjun Liu, Zhigang Ouyang, Zhanjun Lu

**Affiliations:** a School of Life Sciences, Gannan Normal University, Ganzhou, China;; b National Navel Orange Engineering and Technology Research Center, Ganzhou, China

**Keywords:** Eulophidae, mitochondrial genome, *Tamarixia radiata*, phylogenetic analysis

## Abstract

*Tamarixia radiata* plays an important role in biological control of the psyllid *Diaphorina citri* Kuwayama, vector of the huanglongbing (HLB). In this study, we sequenced and analyzed the mitochondrial genome (mitogenome) of *T. radiata*, the first mitogenome of species in the family Eulophidae. This mitogenome was 14,752 bp long and encoded 13 protein-coding genes (PCGs), 22 transfer RNA genes (tRNAs) and two ribosomal RNA unit genes (rRNAs). All 13 PCGs were initiated by the ATN (ATG, ATT, and ATA) codon. Twelve PCGs terminate with the stop codon TAA or TAG except for *nad1* which end with the incomplete codon T−. Phylogenetic analysis showed that *T. radiata* got together with three Pteromalidae species, indicating the close relationship of Eulophidae and Pteromalidae.

*Tamarixia radiata* (Waterston) is an important natural enemy of the Asian citrus psyllid, *Diaphorina citri* Kuwayama (Hemiptera: Liviidae). *Tamarixia radiata* was successfully imported into many countries to suppress populations of *D. citri* (Mann et al. 2010). Mitochondrial DNA has many characteristics which make it highly useful in studies of molecular evolution, phylogenetics and population genetics (Cameron 2014).

Specimens of *T. radiata* were collected from Puning City, Guangdong Province, China (23°17′N, 116°9′E, October 2018) and were stored in Entomological Museum of Gannan Normal University (Accession number GNU-TR031). The mitogenome sequence of *T. radiata* was generated using Illumina HiSeq 2500 Sequencing System. In total, 7.8 G raw reads were obtained, quality-trimmed, and assembled using MITObim v 1.7 (Hahn et al. 2013). By comparison with the homologous sequences of other Chalcidoidea species from GenBank, the mitogenome of *T. radiata* was annotated using software GENEIOUS R8 (Biomatters Ltd., Auckland, New Zealand).

The nearly complete mitogenome of *T. radiata* is 14,752 bp (Genbank accession, MN123622) in length and contains 13 protein-coding genes (PCGs), 22 tRNA genes, two rRNA genes, and one partial non-coding AT-rich region. The region that we failed to sequence in *T. radiata* was the putative control region located between *rrnS* and *nad2*, and similar results were found in other Chalcidoidea species (Su et al. 2016; Zhu et al. 2018; Tang et al. 2019). The overall base composition of the mitogenome was estimated to be A 44.0%, T 41.4%, C 8.0%, and G 6.6%, with a high AT content of 85.4%. Compared with ancestral insect mitochondrial genome, the mitogenome of *T. radiata* exhibit dramatic mitochondrial gene rearrangement, of which are usually found in Chalcidoidea species (Chen et al. 2018; Xiong et al. 2019). All 13 PCGs of *T. radiata* have the conventional ATN start codons for invertebrate mitochondrial PCGs (six ATG, five ATT, and two ATA). Most of the PCGs terminate with the stop codon TAA or TAG, whereas *nad1* end with the incomplete codon T−. The 22 tRNA genes vary from 56 bp (*trnR*) to 70 bp (*trnD* and *trnL1*). Two rRNA genes (*rrnL* and *rrnS*) locate at *trnL1*/*trnA* and *trnA*/*trnV* regions, with the lengths of 1,309 and 754 bp respectively.

To validate the phylogenetic position of *T. radiata*, 13 mitochondrial protein-coding genes sequences were extracted from the mitochondrial DNA sequences of 17 closely related taxa of Chalcidoidea. Phylogenetic tree was constructed using the maximum-likelihood method through raxmlGUI 1.5 (Silvestro and Michalak 2012). Monophyly of the superfamily Chalcidoidea was strongly supported (BS, Bootstrap support value = 100), which was consistent with the previous studies (Chen et al. 2018; Xiong et al. 2019) (Figure 1). The newly sequenced species *T. radiata* got together with three Pteromalidae species (*Pteromalus puparum*, *Philotrypesis* sp. and *Philotrypesis pilosa*) with high support value (BS = 99), indicating the close relationship of Eulophidae and Pteromalidae. In conclusion, the mitogenome of *T. radiata* is decoded in this study and can provide essential and important DNA molecular data for further phylogenetic and evolutionary analysis of Chalcidoidea.

**Figure 1.**
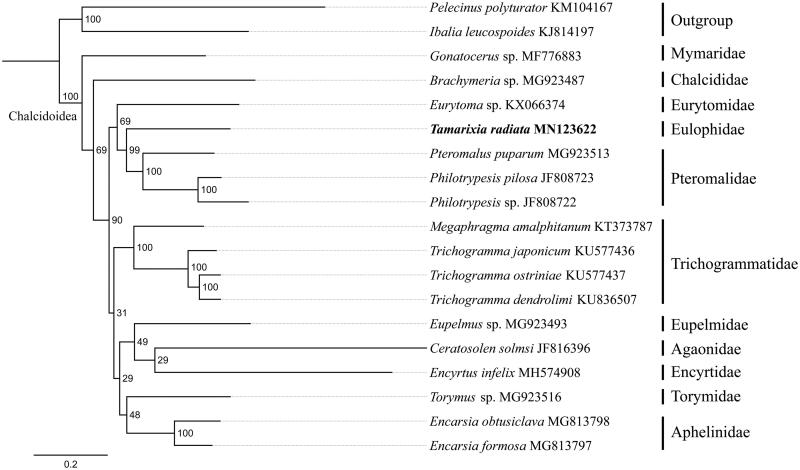
Phylogenetic relationships based on the 13 mitochondrial protein-coding genes sequences inferred from RaxML. Numbers on branches are Bootstrap support values (BS).
